# Neuroprotective effects of gallic acid in a rat model of traumatic brain injury: behavioral, electrophysiological, and molecular studies

**DOI:** 10.22038/IJBMS.2018.29639.7165

**Published:** 2018-10

**Authors:** Mohammad Ali Mirshekar, Alireza Sarkaki, Yaghoub Farbood, Mohammad Kazem Gharib Naseri, Mohammad Badavi, Mohammad Taghi Mansouri, Abbas Haghparast

**Affiliations:** 1 Department of Physiology, School of Medicine, Ahvaz Jundishapur University of Medical Sciences, Ahvaz, Iran; 2 Department of Physiology, School of Medicine and Clinical Immunology Research Center, Zahedan University of Medical Sciences, Zahedan, Iran; 3 Ahvaz Physiology Research Center, Ahvaz Jundishapur University of Medical Sciences, Ahvaz, Iran; 4 Department of Pharmacology, School of Medicine, Ahvaz Jundishapur University of Medical Sciences, Ahvaz, Iran; 5 Neuroscience Research Center, Shahid Beheshti University of Medical Sciences, Tehran, Iran

**Keywords:** Brain inflammation, Gallic acid, Long-term potentiation Memory, Rat, Traumatic brain injury

## Abstract

**Objective(s):**

Traumatic brain injury (TBI) is one of the main causes of intellectual and cognitive disabilities. Clinically, it is essential to limit the development of cognitive impairment after TBI. In the present study, the neuroprotective effects of gallic acid (GA) on neurological score, memory, long-term potentiation (LTP) from hippocampal dentate gyrus (hDG), brain lipid peroxidation and cytokines after TBI were evaluated.

**Materials and Methods:**

Seventy-two adult male Wistar rats divided randomly into three groups with 24 in each: Veh + Sham, Veh + TBI and GA + TBI (GA; 100 mg/kg, PO for 7 days before TBI induction). Brain injury was made by Marmarou’s method. Briefly, a 200 g weight was fallen down from a 2 m height through a free-falling tube onto the head of anesthetized animal.

**Results:**

Veterinary coma scores (VCS), memory and recorded hDG -LTP significantly reduced in Veh + TBI group at 1 and 24 hr after TBI when compared to Veh + Sham (*P*<0.001), respectively, while brain tissue content of interleukin-1β (IL-1β), IL-6, tumor necrosis factor-α (TNF-α) and malondialdehyde (MDA) were increased significantly (*P*<0.001). Pretreatment of TBI rats with GA improved clinical signs, memory and hDG-LTP significantly (*P*<0.001) compared to Veh + TBI group, while brain tissue content of IL-1β, IL-6, TNF-α and MDA were decreased significantly (*P*<0.001).

**Conclusion:**

Our results propose that GA has neuroprotective effect on memory and LTP impairment due to TBI through decrement of brain lipid peroxidation and cerebral pro-inflammatory cytokines.

## Introduction

Traumatic brain injury (TBI) is a main health problem in world affecting millions of people. Great number of deaths and cases of permanent disability are caused by TBI every year (1, 2). It was suggested that TBI as significant health trouble, leads to a potentially catastrophic weakening medical emergency with poor prediction and long-term disability (3). 

Brain injury due to TBI going on at the moment of impact, which has not appear therapeutic feedback and physiological and pathological reactions activates subsequently, these situations provide a chance for clinical remediation. 

These phenomena include release of excitatory neurotransmitters, production of free radicals and activation of inﬂammatory cytokines that contribute to delayed neuronal cell death, which last for days or months (4). 

It was well established that many disabilities such as locomotor activity, intellectual and cognition are manifested in both short and long-term regardless of the severity of the TBI (5-8). 

Alterations in synaptic role within a neural network and subsequent adjustment of cell action have been theorized as a neuronal substrate for cognition. Electrical discharge designs of a single neuron or a group of neurons encode sensory or suitable signals related to learning and memory (9). The most famous cellular mechanism underlying memory is long term potentiation (LTP), which correspond to activity based on synaptic efficacy (10). 

There is a suggestion that pro-inflammatory cytokines such as interleukin-1β (IL-1β), interleukin-6 (IL-6) and tumor necrosis factor-α (TNF-α) engage in TBI conditions and cerebral ischemic. These cytokines are important mediators of infection-related disorders (11). Initiation of inflammatory pathway delayed responses to TBI. Inflammatory mediators such as transforming growth factor- β (TGF-β) and IL-1β are attenuated by anti-inflammatory factors (12). 

Gallic acid (GA), as a phenolic compound, is a natural product that is used in chemical industries such as dye making and tanning (13). Also, it has a broad range of biological properties such as antioxidant and anti- inflammatory activities (14). GA, as form of gallate, is generally utilized as antioxidant by food supplements and pharmaceutical companies (15). Anti-tyrosine action of GA has been reported (16), so it has a protective effect on brain by increasing antioxidant enzymes and reducing inflammation in cerebral hypo-perfusion (17, 18). 

**Figure 1:**
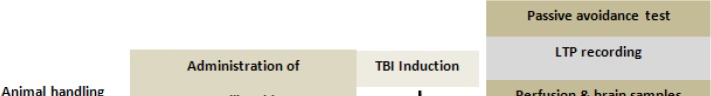
Plan of experimental program and intervals for estimation of various parameters

Herein, this study designed to investigate the effects of GA on memory, LTP, malondialdehyde (MDA) and pro-inflammatory brain cytokines (IL-1β, IL-6 and TNF-α) in response to administration of GA seven days before TBI in male rats.

## Materials and Methods

***Agents***

GA (purity≥98%), Evans blue, Triton-X100 and protease inhibitor cocktail obtained from Sigma-Aldrich Co (St Louis, MO, USA). Tris base, sodium phosphate, sodium chloride, potassium phosphate, potassium chloride and all other chemicals prepared from Merck Company (Darmstadt, Germany).

***Animals***

 Seventy-two adult male Wistar rats (280-320 g) were purchased from animal care and breeding center, Ahvaz Jundishapur University of Medical Sciences (AJUMS), Ahvaz, Iran. They were kept under standard condition, temperature-controlled room (20±2 ^°^C), a 12 hr light/dark cycles and with food and water access *ad libitum*. All experimental procedures were confirmed by AJUMS Ethical Committee (REC. 1392. 363), in agreement with the internationally accepted ethics codes for the care and use of laboratory animals.

***Experimental plan***

 Animals were randomly allocated into three main groups (n=24) as follows: 1) Veh+Sham; sham operated received normal saline (10 ml/kg once daily, gavage) for seven consecutive days before TBI induction. 2) Veh+TBI; TBI group received normal saline (10 ml/kg once daily, gavage) for 7 successive days before TBI induction. GA+TBI; TBI group received GA (100 mg/kg/10 ml once daily, gavage) (19) for same duration before TBI induction.

 GA was dissolved in normal saline; freshly prepared daily (20). Each main group divided into 3 subgroups (n=8) for behavioral, electrophysiological and molecular assessment. Experimental schedule and the intervals for estimation of various parameters are shown in Figure 1. All procedures were completed during light phase between 8:00–12:00 a.m.

***TBI induction***

With the aim of making experimental model of TBI, rats were intubated tracheally under anesthesia. TBI model was made by using a domestic instrument equipped in Ahvaz Physiology Research Center (Ahvaz-Iran) according to Marmarou’s method with some modification (21). Briefly, rats were anesthetized with ketamine/xylazine (90/10 mg/kg, IP), and a 200 g weight was fallen down from a 2 m height through a vertical tube onto the head of animal while a stainless steel disc (r=5 mm & h=3 mm) was attached to its skull. After then, the animal was instantly connected to the small animal ventilator (UGO Basile, Italy) and at the moment of starting the spontaneous breathing, it was disconnected from ventilator and returned to the cage to be cared.

***Assessment of neurological outcome***

Neurological scores were estimated in awakened TBI animals according to veterinary coma scale (VCS) (22). Scoring range was between 3 -15 as follows: the sum of motor response (1-8), visual response (1-4) and respiratory response (1-3). An elevated score was assigned as a better neurological outcome, while lower score indicates injury severity. VCS was assessed at one hour before, and 1, 24 and 48 hr after TBI induction. Based on the VCS score, the severity of head injury can be categorized into mild (13–15), moderate (9 –12), and severe (8 or less).

***Passive avoidance memory evaluation***

In the first subgroup, the passive avoidance task (PAT) was accomplished using shuttle box 48 hr after TBI. The apparatus comprised two illuminate and dark chambers and a slipping door (Borj Sanaat Co, Tehran-Iran). In order to adapt the instrument, rat were located in the light compartment while the sliding door was slipped up and allowed to explore into all parts of device for 5 min. After 10 min, the rat was re-located in the light compartment again facing away from the closed sliding door and the door was opened 10 sec later. Delay of rat entrance into the dark part was recorded as initial latency (IL). As soon as the animals entered the dark compartment, the door was closed and an unescapable electrical foot-shock (50 Hz, 1.2 mA for 3 sec) was delivered through the grid floor with a stimulator. The retention test was performed 24 hr later again using the same paradigm without the foot-shock. Step-through latency (STL) as memory trial was recorded. Cut – off time to avoid into dark section entry was 300 sec (23). 

***Electrophysiological study***

In the 2nd subgroup, two days after TBI induction, heads of anesthetized rats were fixed again in a stereotaxic device with the purpose of electrodes implantation and field excitatory post synaptic potential (fEPSP) recording. The bipolar microelectrodes (metal wire, tungsten wire, 50 µm in diameter, tip separation 1 mm, and stainless steel, 100 µm in diameter, tip separation 500 µm (CFW, USA)) were placed in the granular cells of DG (AP=-3.8 mm from bregma; ML=-2.3 mm; DV=-3.5 mm from dura) and in the perforant pathway (PP) (AP: -7.5 from bregma, ML:-4, DV:-3.9 mm from the dura), respectively (24, 25). The electrodes were pulled down very slowly (0.1 mm/30 sec) from dura to the PP in order to minimize trauma to brain tissue. Establishment of electrodes at the correct position was determined by fEPSP recording (26).

***LTP induction and recording***

Single monopolar pulses (length 50 µs) were passed through stimulating microelectrode at 30 sec intervals. Different intensities were used to record fEPSP with 40% of its maximum amplitude by input/output (I/O) curve. The signal was amplified (×1000), filtered (0.1 Hz-3 KHz), digitized at 2 KHz and stored on a PC. LTP was induced 24 hr later in rats with weak anesthesia following high-frequency stimulation (HFS) including 6 trains of 6 pulses (50 µs) at 400 Hz, 100 ms between each train by repetitive 6 times at a 20 sec interval (e-Probe 4Ch. 12R. Partoye Danesh Co. Iran). LTP was recorded 0.25, 0.5, 1, 3 and 24 hr later. Amplitude (Amp), area under curve (AUC) of population spikes (PSs) and fEPSP slope were measured. The recorded PS was analyzed as percentage increase of baseline fEPSP (23).

***Biochemical assay***

Two days after TBI induction, rats in the 3^rd^ subgroup were anesthetized irreversibly with an overdose of sodium thiopental (80 mg/ kg, IP) and perfused intracardially with normal saline (pH: 7.4) for 1 min to eliminate the intracerebrovascular blood (27, 28). The brains were taken out immediately and stored in -80 ˚C. The whole brain tissues were homogenized in a tissue protein extraction reagent (0.5% Triton X-100, 150 mM/l NaCl, 50 mM/l Tris) and a protease inhibitor cocktail (500 mg tissue per 1 ml of the reagent). Samples were shaken for 90 min, centrifuged (4˚C and 4000 rpm, 15 min) and homogenate supernatant was collected.

***Brain tissue cytokines assessment***

ELISA kit for IL-1β, IL-6 and TNF-α was obtained from eBioscience (San Diego, USA) and assay was performed according to the manufacturer’s recommended procedures. Concentration of cytokines was measured as a picogram of antigen per milligram of brain tissue (29).

***Measurement of brain MDA content***

MDA measurement was performed according to Rao *et al*. 1989 method by using thiobarbituric acid (TBA). Light absorbance of samples was read at 534 nm wave-length by spectrophotometer (Biowave II, UK), and MDA concentration was reported as nM/mg protein (30).

***Statistics***

Data presented as mean±SEM and their normalcy was checked with Kolmogorov–Smirnov test. VCS and LTP 

measures at different times were analyzed by repeated measure (RM)-ANOVA, while the STL data in passive avoidance test and biochemical assays were analyzed by one-way ANOVA followed by Tukey’s *post hoc* test. *P*<0.05 was assigned as significant difference. 

## Results

***Neurological score***

**Figure 2:**
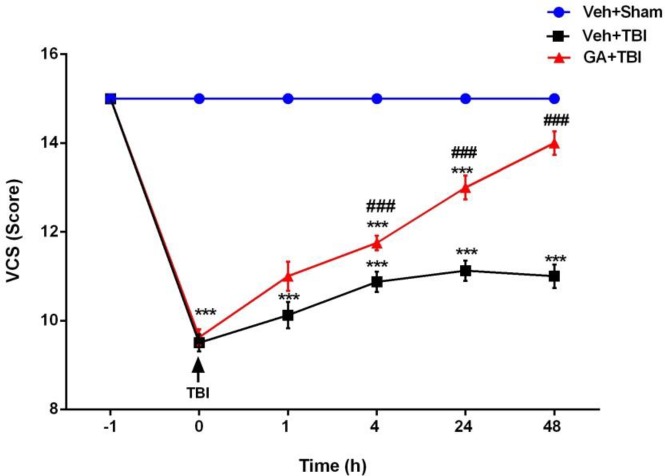
Administration of gallic acid (GA) for 7 days before traumatic brain injury (TBI) on neurological scores after induction of TBI in male rats. Data presented as mean ± SEM, n=72. (*** Significant difference between GA + TBI and vehicle (Veh) + Sham groups (*P<0.001*). ### Significant difference of GA + TBI with Veh + TBI group (*P<0.001*). RM- ANOVA followed by Tukey’s *post hoc* test)

Clinical scores of tested groups are presented in Figure 2. VCS scores revealed a significant reduction in both Veh+TBI and GA+TBI groups at 1 hr after TBI when compared to Veh+Sham (*P*<0.001). Scores in GA+TBI group were significantly greater than Veh+TBI at 4, 24 and 48 hr after TBI (*P*<0.001). 

***Passive avoidance memory***

**Figure 3:**
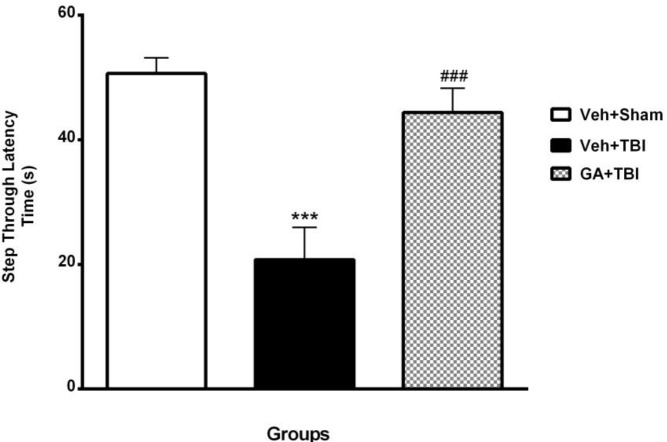
Mean±SEM. of step-through latency (STL) in vehicle (Veh)+Sham, Veh+traumatic brain injury (TBI) and gallic acid (GA)+TBI groups during passive avoidance test (***P<0.001 vs Veh+ ham and ### *P<*0.001 vs TBI group, n=8 in each group, one way ANOVA followed by Tukey’s *post hoc* test)

The STL in Veh+Sham, Veh+TBI and GA+TBI groups are shown in Figure 3. There were no significant alterations in all groups during IL. STL was significantly decreased (*P*<0.001) during memory test at 24 hr after shock delivery to foot paw in Veh+TBI group (20.75±6.71 sec) compared to Veh + Sham (50.62±4.17 sec). STL was significantly improved (*P*<0.001) in GA+TBI group (44.37±8.16 s) in comparison with Veh+TBI. 

***Improvement of electrophysiological indexes by GA***

*PS amplitude*

**Figure 4:**
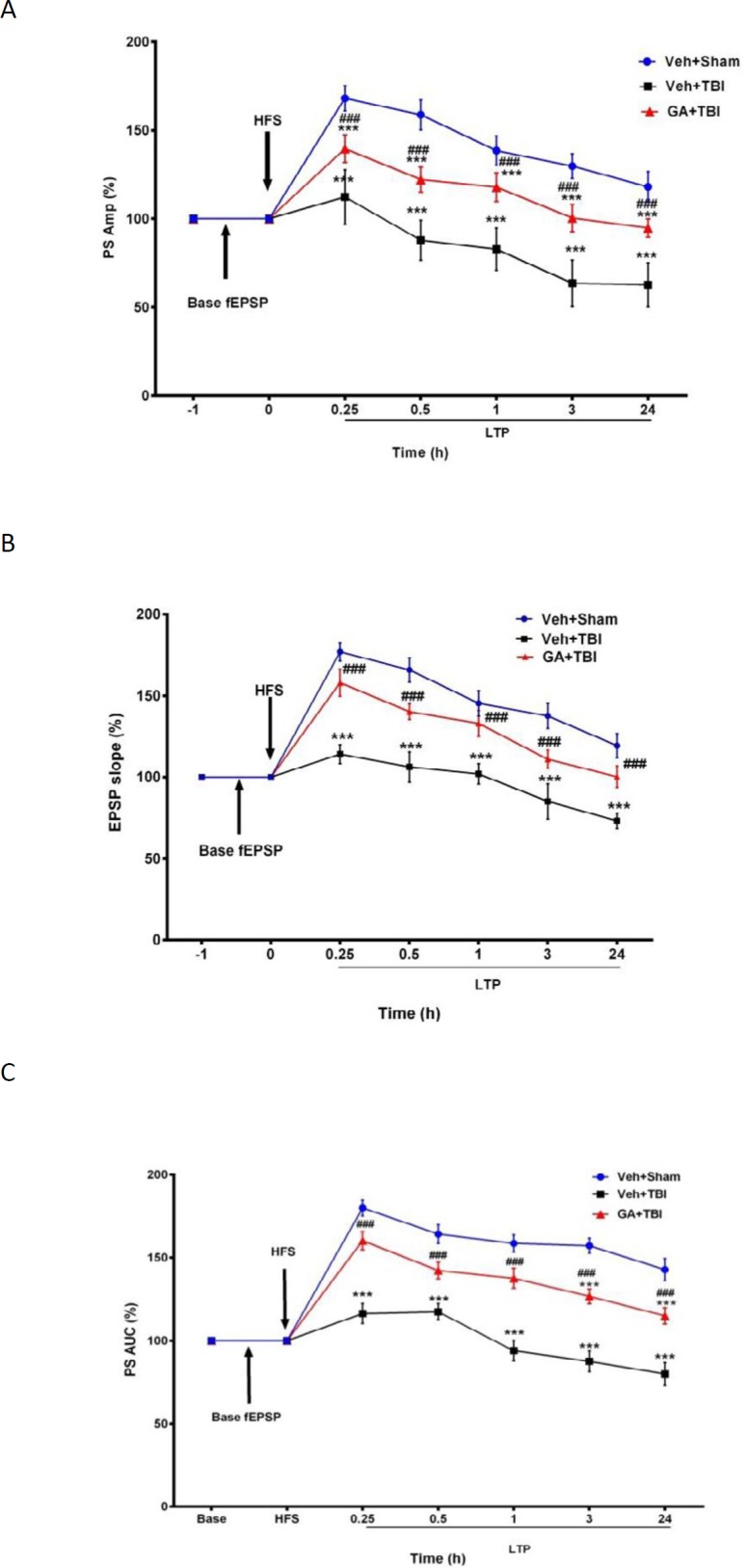
Mean ± SEM of percentages of amplitude (A), area under curve (B) of population spikes (PSs) and slope of fEPSP (C) in different groups during basal fEPSP and long-term potentiation (LTP) recorded from hippocampal DG at 0.25, 0.5, 1, 3, and 24 hr after high frequency stimulation (HFS) to brain PP (Repeated measures two- way ANOVA, followed by HSD *post hoc* test, n=8, ** P<0.01 and *** *P*<0.001, vehicle (Veh)+traumatic brain injury (TBI) and gallic acid (GA)+TBI vs. Veh+Sham, ### *P*<0.001, other groups vs Veh+TBI). Veh + Sham: sham operated received normal saline, Veh+TBI. TBI rats received normal saline, GA+TBI. TBI rats pretreated with 100 mg/kg GA. Base excitatory post synaptic potential (EPSP). EPSP recorded one hour before HFS, LTP 0.25-24. LTP recorded during different times after HFS.was increased significantly (*P*<0.001) at similar times in GA+TBI group when compared to Veh+TBI

As shown in Figure 4A, the PS Amp (mv) reduced significantly (*P*<0.001) in Veh+TBI during all recording times post HFS compared to Veh+Sham group, while it was improved significantly (*P*<0.001) at the same times in GA+TBI group when compared to Veh+TBI. 

***fEPSP slope***

 As shown in Figure 4B, the slope of fEPSP (v/s) in Veh+TBI group was significantly less (*P*<0.001) than Veh+Sham group during all recording times, while it 

***PS AUC***

Figure 4C shows no significant decay in AUC (v_*_s) of population spike in Veh+Sham group, while it decreased significantly in Veh+TBI group during all recording times (*P*<0.001). Pre-TBI treatment with GA caused significant AUC augmentation (*P*<0.001) during all recording times. 

***Decrement of brain IL-*** ***1*** ***β*** ***content by GA ***

**Figure 5:**
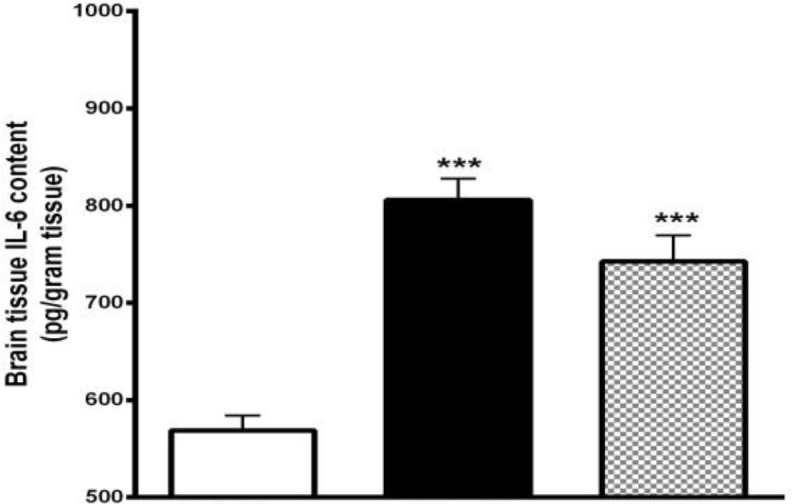
Effect of gallic acid (GA) administration for 7 days before traumatic brain injury (TBI) on brain interleukin-1β (IL-1β) content (pg/ml) in male rats (n= 8 in each group). *P<0.05, ***P<0.001, for vehicle (Veh)+TBI and GA+TBI vs. Veh + Sham, ##* P*<0.01, for GA + TBI vs. Veh+TBI. One way ANOVA followed by Tukey’s *post hoc *test

The effect of pre-TBI administration of GA for 7 days on IL-1β of brain tissue at 48 hr after TBI is shown in Figure 5. IL-1β content in Veh+TBI animals (185.37± 7.51 pg/ml) was significantly greater than Veh + Sham group (120.46±5.50 pg/ml) (*P*<0.001). Pretreatment with GA caused a significant decrease (*P*<0.01) of IL-1β content (149.37±6.54 pg/ml) compared to Veh+TBI group. 

**Figure 6:**
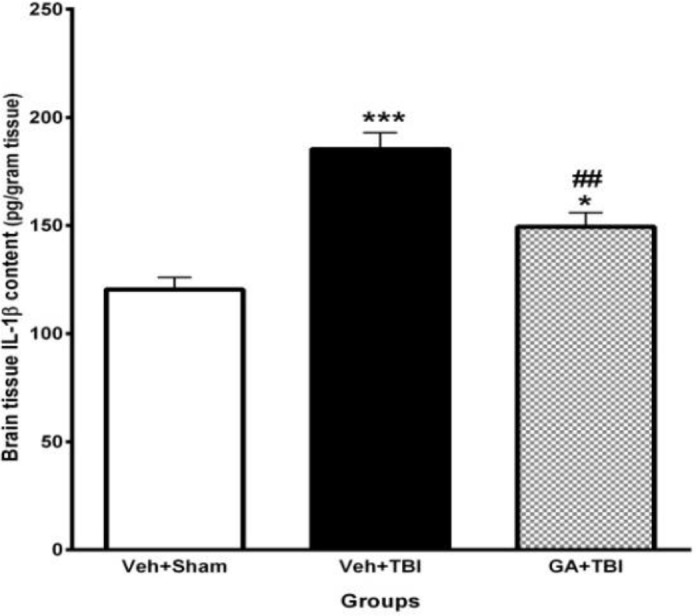
Effect of gallic acid (GA) administration for 7 days before traumatic brain injury (TBI) on brain interleukin-6 (IL-6) content (pg/ml) in male rats (n= 8 in each group). **P<0.01, ***P<0.001, for vehicle (Veh)+TBI and GA+TBI vs. Veh + Sham, # *P*<0.05, for GA+TBI vs. Veh+TBI. One way ANOVA followed by Tukey’s *post hoc* test

**Figure 7:**
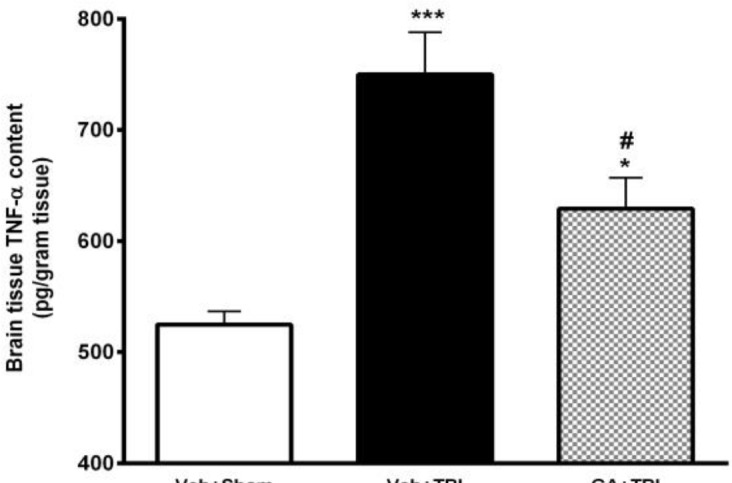
Effect of gallic acid (GA) administration for 7 days before traumatic brain injury (TBI) on brain levels of tumor necrosis factor-α (TNF-α) (pg/ml) in male rats (n= 8 in each group). ***P<0.001, for vehicle (Veh)+TBI vs. Veh + Sham, ## *P*<0.01, for GA+TBI vs. Veh+TBI. One way ANOVA followed by Tukey’s* post hoc* test

**Figure 8:**
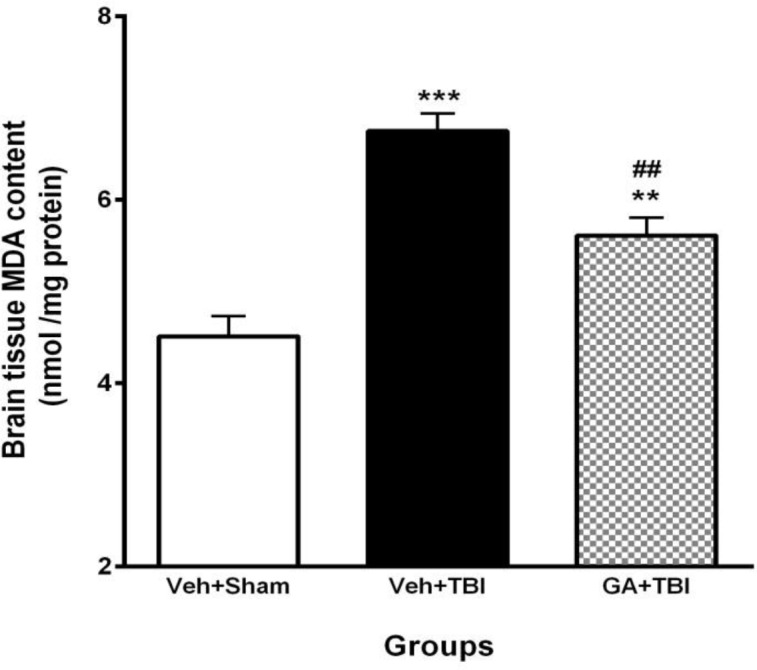
Effect of gallic acid (GA) administration for 7 days before traumatic brain injury (TBI) on brain malondialdehyde (MDA) content (nM/mg protein) in male rats (n=8 in each group). **P<0.01, ***P< 0.001, for vehicle (Veh)+TBI vs. Veh+Sham, ## *P*<0.01, for GA+TBI vs. Veh + TBI. One way ANOVA followed by Tukey’s* post hoc *test

***Decrement of brain IL- 6 content by GA***

The effect of GA administration for 7 days before TBI on the brain content of IL-6 at 48 hr after TBI was shown in Figure 6. IL-6 content in Veh+TBI animals (805.88±21.85 pg/ml) was significantly higher than Veh+Sham group (568.87±15.22 pg/ml) (*P<0.001*). Pretreatment with GA caused a significant decrease (*P<0.01*) of IL-6 content (717.5±30.22 pg/ml) compared to Veh+TBI group. 

***Decrement of brain TNF-*** ***α*** ***content by GA ***

The effect of GA administration for 7 days before TBI on the brain content of TNF-α at 48 hr after TBI was shown in Figure 7. TNF-α content in Veh+TBI animals (687.46±29.34 pg/ml) was significantly higher than Veh+Sham group (524.92±11.82 pg/ml) (*P*<0.001). Pretreatment with GA caused a significant decrease (*P*<0.01) of TNF-α content (591.66±8.73 pg/ml) compared to Veh+TBI group. 

***Decrement of brain MDA content by GA***

Pretreatment effect of GA on the brain content of MDA at 48 hr after TBI was shown in Figure 8. MDA content in Veh+TBI animals (6.75±0.19) was significantly higher than Veh + Sham group (4.51±0.22) (*P*<0.001). Administration of GA for 7 days before TBI caused a significant decrement (*P*<0.01) of MDA content (5.61±0.19) compared to Veh+TBI group. 

## Discussion

In the present study, cognitive impairment was achieved by TBI, which was shown by decreasing STL in passive avoidance test at 48 hr after TBI. This result was in agreement with the previous investigation (31, 32). It has been recognized that pretreatment with GA (100 mg/kg, PO) seven days pre-TBI could improve memory, evidenced by longer STL at 48 hr after TBI. Furthermore, this effect was associated with enhancement of neurological scores. 

The severity of neuroanatomical, neurochemical, and neurological outcomes is related to strictness of the insult measured in depth/velocity for impact models determines. Experimental brain trauma results in both sensorimotor and cognitive behavioral discrepancies (33-37). Our findings are consistent with other investigators results.

In passive avoidance test, the rats in the first trial obtain information to enter into dark chamber. These results in painful experience of electric shock, and the cognitive ability of the animals were reflected by avoidance to entry (a judgment based on successful retention and recall of the acquired information). When injured animals (such as Veh+TBI group in our experiment) were checked in retention trial, there was no increase in STL, which reflects cognitive impairment. 

Cortical impact injury is renowned for its ability to model for impaired learning and memory. TBI results in hippocampal degeneration, with grade of atrophy related to severity of brain damage (31). Hippocampus, as an important area of the brain involved in cognition, is very sensitive to injury (38). Hippocampus is characterized by a low capillary mass compared to its other subdivisions (39). Hippocampal pyramidal neurons are more vulnerable and die due to TBI. So, its damage will be observed a few days after injury in rat. Accordingly, hippocampal synaptic transmission and plasticity could be deteriorated and disrupted by TBI (40, 41). Pretreatment of TBI rats with GA causes latency enhancement in a retention trial of avoidance memory; these findings have been observed by other investigators that used different doses of GA in experimental Parkinson’s disease induced by 6-hydroxydopamine (19).

Represented data indicate that GA pretreatment increased amplitude, AUC of LTP and slope of fEPSP recorded from DG following HFS. Furthermore, TBI removed PS record from DG area, which is consistent with some earlier findings (42-44).

Pivotal role of calcium ion in signaling in all tissues has been detected and it is vital for normal synaptic transmission in CNS. Complicated processes regulate calcium release, and intracellular systems are concerned for responding to a calcium stimulus. Both brain injury and synaptic plasticity are mediated by calcium signaling processes. N-methyl, D-aspartate (NMDA) and α-amino-3-hydroxy-5-methyl-4-isoxazolepropionic acid (AMPA) receptors of glutamate activation lead to calcium signaling and homeostasis (45). Calcium homeostasis obliterated by TBI and lengthened rise of calcium amount lead to some processes like extreme glutamate release and activation of NMDA receptors, creation of free radicals, outward flow of potassium ions, cellular swelling and activation of cytokines such as TNF-α and IL-1β (46). 

Tate and Bigler suggested that mentioned disrupted mechanisms achieved by TBI, probably resulted from increasing severe inflammatory responses as well as oxidative stress in rat brains (31). Based on our results, TBI amplified pro-inflammatory cytokines, which confirmed by previous investigations. Moreover, some other investigations showed that release of pro-inflammatory cytokines provoke delayed response to TBI (47, 48). It seems that promoting the inflammation might be mediated by augmentation of brain cytokines. In current study, we showed that brain content of pro-inflammatory cytokines such as IL-1β, IL-6 and TNF-α increased after TBI. Increment of TNF-α level following TBI has been reported to be an important reason for brain parenchyma response to the injury (49). 

In addition, many published data confirmed that these cytokines are increased during inflammation (50-52). Our results have shown that GA pretreatment decreased brain IL-1β, 48 hr after the TBI. Several lines of studies have reported that changes in the contents of cytokines occur in different models of injury such as frontal brain ischemia (29), injuries induced by fluid percussion (53), spinal cord injury (54) and experimental TBIs (55). Brain cytokines contents change 30 minutes following TBI and last for 24 hr (12). Current data showed that pretreatment with GA reduced IL-6 and TNF-α in brain 48 hr after TBI. Elevation of IL-6 due to TBI induces neuronal death and blood–brain barrier disruption accompanied by severe clinical disorders (51). 

The beneficial therapeutic action of phenolic compounds is related to their anti-inflammatory, antioxidant capacity and free radical scavenging activity (56). GA, as a polyhydroxyphenolic compound, is one of the major bioactive compounds isolated and purified from number of plants (57-59). Various pharmacological activities of GA such as anticancer (60) and antioxidant function (60) have been reported. This compound has also been described as an excellent free radical scavenger (61). TBI might induce a significant up-regulation of TNF-α, IL-6 and NF-kB (nuclear factor kappa-light-chain-enhancer of activated B cells) in animal cell types and is involved in cellular responses to stimuli such as stress, cytokines and free radicals in the rat tissues. NF-kB binding activity was significantly increased from 3 hr to 7 days post-injury, with the maximum at 72 hr. Moreover, TNF-α and IL-6 were significantly increased after TBI and remained elevated on Day 7 post-injury. In addition, there was a positive connection between the expression of NF-kB and the pro-inflammatory cytokines TNF-α and IL-6 (62). In an additional study, it has been established that GA can inhibit TNF-α induced inflammatory role of NF-kB (63). According to previous researches and on base of our results, we hypothesized that pretreatment with GA recovers cerebral inflammation in rats because of its anti-inflammatory and antioxidative actions.

## Conclusion

In summary, our results propose that release of cytokines such as IL-1β, IL-6 and TNF-α and also MDA as lipid peroxidation index in brain tissue following TBI cause cognitive impairment and LTP insufficiencies. We found that administration of GA before moderate TBI induction reversed the contents of IL-1β, IL-6, and TNF-α and brain tissue MDA, thereby improves memory and LTP indexes. Further studies are necessary to clarify more neuroprotective mechanisms of GA.

## References

[1] Brooks JC, Strauss DJ, Shavelle RM, Paculdo DR, Hammond FM, Harrison-Felix CL (2013). Long-term disability and survival in traumatic brain injury: results from the national institute on disability and rehabilitation research model systems. Arch Phys Med Rehabil.

[2] Faul M, Xu L, Wald M, Coronado VG (2010). National Center for Injury Prevention and Control. Traumatic brain injury in the United States: emergency department visits, hospitalizations and deaths 2002–2006.

[3] Wang KK, Larner SF, Robinson G, Hayes RL (2006). Neuroprotection targets after traumatic brain injury. Curr Opin Neurol.

[4] Kumar A, Loane DJ (2012). Neuroinflammation after traumatic brain injury: opportunities for therapeutic intervention. Brain Behav Immun.

[5] Ozen LJ, Fernandes MA (2012). Slowing down after a mild traumatic brain injury: a strategy to improve cognitive task performance. Arch Clin Neuropsychol.

[6] Trivedi MA, Ward MA, Hess TM, Gale SD, Dempsey RJ, Rowley HA (2007). Longitudinal changes in global brain volume between 79 and 409 days after traumatic brain injury: relationship with duration of coma. J Neurotrauma.

[7] Werner C, Engelhard K (2007). Pathophysiology of traumatic brain injury. Br J Anaesth.

[8] Greenberg G, Mikulis DJ, Ng K, DeSouza D, Green RE (2008). Use of diffusion tensor imaging to examine subacute white matter injury progression in moderate to severe traumatic brain injury. Arch Phys Med Rehabil.

[9] Deadwyler SA, Bunn T, Hampson RE (1996). Hippocampal ensemble activity during spatial delayed-nonmatch-to-sample performance in rats. J Neurosci.

[10] Collingridge G, Bliss T (1995). Memories of NMDA receptors and LTP. Trends Neurosci.

[11] Sirota L, Shacham D, Punsky I, Bessler H (2001). Ibuprofen affects pro-and anti-inflammatory cytokine production by mononuclear cells of preterm newborns. Biol Neonate.

[12] Sarkaki AR, Khaksari Haddad M, Soltani Z, Shahrokhi N, Mahmoodi M (2013). Time-and dose-dependent neuroprotective effects of sex steroid hormones on inflammatory cytokines after a traumatic brain injury. J Neurotrauma.

[13] Qi FH, Jing TZ, Wang ZX, Zhan YG (2009). Fungal endophytes from Acer ginnala Maxim: isolation, identification and their yield of gallic acid. Lett Appl Microbiol.

[14] Kratz JM, Andrighetti-Frohner CR, Leal PC, Nunes RJ, Yunes RA, Trybala E (2008). Evaluation of anti-HSV-2 activity of gallic acid and pentyl gallate. Biol Pharm Bull.

[15] Kratz JM, Andrighetti-Frohner CR, Kolling DJ, Leal PC, Cirne-Santos CC, Yunes RA (2008). Anti-HSV-1 and anti-HIV-1 activity of gallic acid and pentyl gallate. Mem Inst Oswaldo Cruz.

[16] Kim YJ (2007). Antimelanogenic and antioxidant properties of gallic acid. Biol Pharm Bull.

[17] Mansouri MT, Farbood Y, Sameri MJ, Sarkaki A, Naghizadeh B, Rafeirad M (2013). Neuroprotective effects of oral gallic acid against oxidative stress induced by 6-hydroxydopamine in rats. Food Chem.

[18] Sarkaki A, Farbood Y, Gharib-Naseri MK, Badavi M, Mansouri MT, Haghparast A (2015). Gallic acid improved behavior, brain electrophysiology, and inflammation in a rat model of traumatic brain injury. Can J Physiol Pharmacol.

[19] Mansouri MT, Farbood Y, Sameri MJ, Sarkaki A, Naghizadeh B, Rafeirad M (2013). Neuroprotective effects of oral gallic acid against oxidative stress induced by 6-hydroxydopamine in rats. Food Chem.

[20] Manach C, Williamson G, Morand C, Scalbert A, Rémésy C (2005). Bioavailability and bioefficacy of polyphenols in humans I Review of 97 bioavailability studies. Am J Clin Nutr.

[21] O’Connor CA, Cernak I, Vink R (2005). Both estrogen and progesterone attenuate edema formation following diffuse traumatic brain injury in rats. Brain Res.

[22] King DR, Cohn SM, Proctor KG (2004). Changes in intracranial pressure, coagulation, and neurologic outcome after resuscitation from experimental traumatic brain injury with hetastarch. Surgery.

[23] Lashgari R, Motamedi F, Zahedi Asl S, Shahidi S, Komaki A (2006). Behavioral and electrophysiological studies of chronic oral administration of L-type calcium channel blocker verapamil on learning and memory in rats. Behav Brain Res.

[24] Paxinos G, Watson C (2006). The rat brain in stereotaxic coordinates: hard cover edition.

[25] Roohbakhsh A, Moghaddam AH, Massoudi R, Zarrindast MR (2007). Role of dorsal hippocampal cannabinoid receptors and nitric oxide in anxiety like behaviours in rats using the elevated plus-maze test. Clin Exp Pharmacol Physiol.

[26] Sarkaki A, Rafieirad M, Hossini SE, Farbood Y, Motamedi F, Mansouri SMT (2013). Improvement in memory and brain long-term potentiation deficits due to permanent hypoperfusion/ischemia by grape seed extract in rats. Iran J Basic Med Sci.

[27] Dubal DB, Wise PM (2001). Neuroprotective effects of estradiol in middle-aged female rats 1. Int J Endocrinol.

[28] Holmin S, Höjeberg B (2004). In situ detection of intracerebral cytokine expression after human brain contusion. Neurosci Lett.

[29] Taupin V, Toulmond S, Serrano A, Benavides J, Zavala F (1993). Increase in IL-6, IL-1 and TNF levels in rat brain following traumatic lesion: Influence of pre-and post-traumatic treatment with Ro5 4864, a peripheral-type (p site) benzodiazepine ligand. J Neuroimmunol.

[30] Rao B, Soufir J, Martin M, David G (1989). Lipid peroxidation in human spermatozoa as relatd to midpiece abnormalities and motility. Gamete Res.

[31] Tate DF, Bigler ED (2000). Fornix and hippocampal atrophy in traumatic brain injury. Learn Mem.

[32] Mashhadizadeh S, Farbood Y, Dianat M, Khodadadi A, Sarkaki A (2017). Therapeutic effects of ellagic acid on memory, hippocampus electrophysiology deficits, and elevated TNF-alpha level in brain due to experimental traumatic brain injury. Iran J Basic Med Sci.

[33] McIntosh T, Vink R, Noble L, Yamakami I, Fernyak S, Soares H (1989). Traumatic brain injury in the rat: characterization of a lateral fluid-percussion model. Neuroscience.

[34] Lyeth B, Jenkins L, Hamm R, Dixon C, Phillips L, Clifton G (1990). Prolonged memory impairment in the absence of hippocampal cell death following traumatic brain injury in the rat. Brain Res.

[35] Hamm RJ, Lyeth BG, Jenkins LW, O’Dell DM, Pike BR (1993). Selective cognitive impairment following traumatic brain injury in rats. Behav Brain Res.

[36] Hicks R, Smith D, Lowenstein D, Marie RS, McIntosh T (1993). Mild experimental brain injury in the rat induces cognitive deficits associated with regional neuronal loss in the hippocampus. J Neurotrauma.

[37] Bramlett HM, Green EJ, Dietrich WD, Busto R, Globus MY, Ginsberg MD (1995). Posttraumatic brain hypothermia provides protection from sensorimotor and cognitive behavioral deficits. J Neurotrauma.

[38] Farkas E, Luiten PG, Bari F (2007). Permanent, bilateral common carotid artery occlusion in the rat: a model for chronic cerebral hypoperfusion-related neurodegenerative diseases. Brain Res Rev.

[39] Cavaglia M, Dombrowski SM, Drazba J, Vasanji A, Bokesch PM, Janigro D (2001). Regional variation in brain capillary density and vascular response to ischemia. Brain Res.

[40] Yaka R, Biegon A, Grigoriadis N, Simeonidou C, Grigoriadis S, Alexandrovich AG (2007). D-cycloserine improves functional recovery and reinstates long-term potentiation (LTP) in a mouse model of closed head injury. FASEB J.

[41] Farbood Y, Sarkaki A, Dianat M, Khodadadi A, Haddad MK, Mashhadizadeh S (2015). Ellagic acid prevents cognitive and hippocampal long-term potentiation deficits and brain inflammation in rat with traumatic brain injury. Life Sci.

[42] Miyazaki S, Katayama Y, Lyeth B, Jenkins L, Dewitt D, Goldberg S (1992). Enduring suppression of hippocampal long-term potentiation following traumatic brain injury in rat. Brain Res.

[43] Sanders MJ, Sick TJ, Perez-Pinzon MA, Dietrich WD, Green EJ (2000). Chronic failure in the maintenance of long-term potentiation following fluid percussion injury in the rat. Brain Res.

[44] Albensi BC, Sullivan PG, Thompson MB, Scheff SW, Mattson MP (2000). Cyclosporin ameliorates traumatic brain-injury-induced alterations of hippocampal synaptic plasticity. Exp Neurol.

[45] Choi DW (1995). Calcium: still center-stage in hypoxic-ischemic neuronal death. Trends in Neurosci.

[46] Albensi BC (2001). Models of brain injury and alterations in synaptic plasticity. J Neurosci Res.

[47] de Vries HE, Kuiper J, de Boer AG, Van Berkel TJ, Breimer DD (1997). The blood-brain barrier in neuroinflammatory diseases. Pharmacol Rev.

[48] del Zoppo GJ, Hallenbeck JM (2000). Advances in the vascular pathophysiology of ischemic stroke. Thrombo Res.

[49] Rostworowski M, Balasingam V, Chabot S, Owens T, Yong VW (1997). Astrogliosis in the neonatal and adult murine brain post-trauma: elevation of inflammatory cytokines and the lack of requirement for endogenous interferon-γ. J Neurosci.

[50] Kamm K, Vanderkolk W, Davis AT, Lawrence C, Jonker M (2006). The effect of traumatic brain injury upon the concentration and expression of interleukin-1 and interleukin-10 in the rat. J Trauma.

[51] Lenzlinger PM, Morganti-Kossmann MC, Laurer HL, McIntosh TK (2001). The duality of the inflammatory response to traumatic brain injury. Mol Neurobiol.

[52] Chen G, Shi J, Jin W, Wang L, Xie W, Sun J (2008). Progesterone administration modulates TLRS/NF-κB signaling pathway in rat brain after cortical contusion. Ann Clin Lab Sci.

[53] Fan L, Young PR, Barone FC, Feuerstein GZ, Smith DH, McIntosh TK (1996). Experimental brain injury induces differential expression of tumor necrosis factor-α mRNA in the CNS. Brain Res Mol Brain Res.

[54] Stein DG, Wright DW, Kellermann AL (2008). Does progesterone have neuroprotective properties?. Ann Emerg Med.

[55] McIntosh TK, Juler M, Wieloch T (1998). Novel pharmacologic strategies in the treatment of experimental traumatic brain injury: 1998. J Neurotrauma.

[56] Cai Y, Luo Q, Sun M, Corke H (2004). Antioxidant activity and phenolic compounds of 112 traditional Chinese medicinal plants associated with anticancer. Life Sci.

[57] De Beer D, Joubert E, Gelderblom WC, Manley M (2003). Antioxidant activity of South African red and white cultivar wines: free radical scavenging. J Agric Food Chem.

[58] Sun J, Chu YF, Wu X, Liu RH (2002). Antioxidant and antiproliferative activities of common fruits. J Agric Food Chem.

[59] Wolfe K, Wu X, Liu RH (2003). Antioxidant activity of apple peels. J Agric Food Chem.

[60] Yang HL, Chang WH, Chia YC, Huang CJ, Lu FJ, Hsu HK (2006). Toona sinensis extracts induces apoptosis via reactive oxygen species in human premyelocytic leukemia cells. Food Chem Toxicol.

[61] Isuzugawa K, Inoue M, Ogihara Y (2001). Catalase contents in cells determine sensitivity to the apoptosis inducer gallic acid. Biol Pharm Bull.

[62] Hang CH, Shi JX, Li JS, Li WQ, Wu W (2005). Expressions of intestinal NF-κB, TNF-α, and IL-6 following traumatic brain injury in rats. J Surg Res.

[63] Morais MC, Luqman S, Kondratyuk TP, Petronio MS, Regasini LO, Silva DH (2010). Suppression of TNF-α induced NFκB activity by gallic acid and its semi-synthetic esters: possible role in cancer chemoprevention. Nat Prod Res.

